# Computationally-guided drug repurposing enables the discovery of kinase targets and inhibitors as new schistosomicidal agents

**DOI:** 10.1371/journal.pcbi.1006515

**Published:** 2018-10-22

**Authors:** Sandra Giuliani, Arthur C. Silva, Joyce V. V. B. Borba, Pablo I. P. Ramos, Ross A. Paveley, Eugene N. Muratov, Carolina Horta Andrade, Nicholas Furnham

**Affiliations:** 1 Department of Pathogen Molecular Biology, London School of Hygiene and Tropical Medicine, London, United Kingdom; 2 LabMol - Laboratory for Molecular Modeling and Drug Design, Faculdade de Farmácia, Universidade Federal de Goiás - UFG, Goiânia, Goiás, Brazil; 3 Instituto Gonçalo Moniz, Fundação Oswaldo Cruz (FIOCRUZ), Salvador, Bahia, Brazil; 4 Laboratory for Molecular Modeling, Division of Chemical Biology and Medicinal Chemistry, University of North Carolina, Chapel Hill, North Carolina, United States of America; 5 Department of Chemical Technology, Odessa National Polytechnic University, Odessa, Ukraine; 6 Laboratory of Tropical Diseases, Department of Genetics, Evolution, Microbiology and Immunology, Institute of Biology, University of Campinas, Campinas, São Paulo, Brazil; University of Houston, UNITED STATES

## Abstract

The development of novel therapeutics is urgently required for diseases where existing treatments are failing due to the emergence of resistance. This is particularly pertinent for parasitic infections of the tropics and sub-tropics, referred to collectively as neglected tropical diseases, where the commercial incentives to develop new drugs are weak. One such disease is schistosomiasis, a highly prevalent acute and chronic condition caused by a parasitic helminth infection, with three species of the genus *Schistosoma* infecting humans. Currently, a single 40-year old drug, praziquantel, is available to treat all infective species, but its use in mass drug administration is leading to signs of drug-resistance emerging. To meet the challenge of developing new therapeutics against this disease, we developed an innovative computational drug repurposing pipeline supported by phenotypic screening. The approach highlighted several protein kinases as interesting new biological targets for schistosomiasis as they play an essential role in many parasite’s biological processes. Focusing on this target class, we also report the first elucidation of the kinome of *Schistosoma japonicum*, as well as updated kinomes of *S*. *mansoni* and *S*. *haematobium*. In comparison with the human kinome, we explored these kinomes to identify potential targets of existing inhibitors which are unique to *Schistosoma* species, allowing us to identify novel targets and suggest approved drugs that might inhibit them. These include previously suggested schistosomicidal agents such as bosutinib, dasatinib, and imatinib as well as new inhibitors such as vandetanib, saracatinib, tideglusib, alvocidib, dinaciclib, and 22 newly identified targets such as CHK1, CDC2, WEE, PAKA, MEK1. Additionally, the primary and secondary targets in *Schistosoma* of those approved drugs are also suggested, allowing for the development of novel therapeutics against this important yet neglected disease.

## Author summary

The rise of resistance through the intensive use of drugs targeted to treat specific infectious diseases means that new therapeutics are continually required. Diseases common in the tropics and sub-tropics, classified as neglected tropical diseases, suffer from a lack of new drug treatments due to the difficulty in developing new drugs and the lack of market incentive. One such disease is schistosomiasis, a major human helminth disease caused by worms from the genus *Schistosoma*. It is currently treated by a 40-year old drug, praziquantel, but its widespread use has led to signs of drug-resistance emerging, with no alternative effective treatments available. To meet this challenge, we have developed an innovative computational drug repurposing pipeline supported by experimental phenotypic screening. Protein kinases emerged from our pipeline as interesting new biological targets. Given that many human kinase inhibitors have been successfully applied specially in cancer therapy and kinases have conserved structures and functions, we also undertook a detailed analysis of the kinases present in all infective *Schistosoma* species and human host. This allowed identification of new *Schistosoma*-specific kinase targets and suggest approved drugs to be used for treating schistosomiasis as well as opening new avenues to treat this neglected disease.

## Introduction

Morbidity due to helminth infections affects over one billion people, particularly in marginalized, resource-constrained regions of the world, propagating a vicious circle of poverty, decreased productivity, and inadequate socioeconomic development. One of the most prevalent helminth infections is schistosomiasis, an acute and chronic disease caused by parasitic worms of the genus *Schistosoma*. This debilitating disease is widespread in lower and middle income countries of the tropics and sub-tropics, with more than 200 million people affected worldwide and an estimated 800 million at risk [1, 2]. It is second only to malaria in terms of prevalence and social and economic impact. Three main species infect people often during routine agricultural, domestic, occupational and recreational activities that expose them to infested water: *S*. *mansoni* (South America and sub-Saharan Africa), *S*. *haematobium* (Africa) and *S*. *japonicum* (South-East Asia). Lack of hygiene and certain play habits of school-aged children such as swimming or fishing in infested water make them especially vulnerable to infection. In the Americas, Brazil has the largest endemic area and accounts for 95% of cases of *S*. *mansoni* in the region, with severe cases still occurring [3]. Currently there is only one 40-year-old drug, praziquantel (PZQ), which is effective against all forms of human schistosomiasis. Though in many respects it is still a useful antischistosomal drug, it has low efficacy against the juvenile stage (2–4 weeks post infection) of schistosomes, a limitation that has significant impact on the efficacy of mass drug administration (MDA) programs in endemic areas where reinfection rates are high [4]. In addition, WHO is currently recommending PZQ for MDA and there are concerns that this could lead to resistance and therapeutic failure [5].

Schistosomiasis is neglected by the pharmaceutical industry, yet it is still an important disease that continues to impact the poorest and most vulnerable individuals in society. As its treatment relies on a single available drug, PZQ, with a propensity for resistance to develop to it, discovery of novel antischistosomal drugs is of paramount importance. An important starting point for the discovery for new antischistosomal therapeutics is the identification of novel targets. One route to this is through drug repurposing, also known as drug repositioning or re-profiling [6, 7]. It is the new application for an existing drug to a different disease and offers a highly attractive means to develop novel therapeutics for diseases where current treatments are no longer as effective or do not yet exist [8]. It has two major advantages compared to *de novo* drug discovery, namely reduced development time of a new chemical entity and high probability of success as in most cases the repurposing candidate has already gone through many stages of development for its original therapeutic use [9]. These aspects make it of interest in neglected disease drug discovery where market incentives are generally low.

Several methods have been developed for repurposing drugs mostly within species but also between species. Some of the most straightforward methods use sequences to identify gene signatures, while more sophisticated methods combine sequence with protein structural information. For example, off-target effects can be identified based on target-ligand complexes linked by homology based on whole-sequence alignments to potential new targets [10]. Complete protein similarity does not guarantee binding site similarity, thus new methods have been developed that specifically investigate the proposed binding site, and can be augmented with molecular docking and molecular dynamics simulations [11–13]. Using chemical similarities of the ligand, rather than the target, is another widely used technique with many chemical descriptors being developed and used in a variety of search algorithms [14, 15]. Most of these approaches have been applied in the context of drug repurposing for human targets. In the context of repurposing for neglected tropical disease (NTD) extra challenges are brought, as a drug repositioned for an NTD will inevitably have to be active across different species, meaning the identification of evolutionary related potential targets might be more challenging, yet still be different enough from the host to minimise off target side effects.

Several efforts have been made in repurposing drugs for schistosomiasis. This has included using phenotypic screens of libraries of current Food and Drug Administration (FDA) approved drugs [16–18] as well as single target based methods [16, 19–21] and chemogenomics approaches [6]. The target-based approaches tend to be formulated around a single putative target identified from fundamental insights of helminth biology. This leads to challenges of validating the target and drug efficacy *in vivo*. Our own past target-based approaches have used QSAR-based virtual screening against *Schistosoma mansoni* thioredoxin glutathione reductase (*Sm*TGR) combined with phenotypic screening [22, 23]. Phenotypic screening has arguably been more successful in identifying drugs that work on the parasite [24], including *in vivo* verification in an infectious mouse model. These studies often lack the knowledge of the drug target, which can hinder further drug development and optimisation.

One of the dominant classes of drugs open for repurposing are kinase inhibitors due to the huge effort to develop inhibitors targeting human kinases, successfully applied in cancer therapy [25]. Kinomes of two (*S*. *mansoni* and *S*. *hematobium*) of the three major infective *Schistosoma* species of humans have been proposed [26–28]. In evolutionary terms, the distance between *Schistosoma* and *H*. *sapiens* is small [29] but large enough for difference to exist, thus similarity can be exploited from a drug discovery point of view as a human kinase inhibitor could display activity against a helminth orthologue as well as act as a starting point to explore new chemical series [16, 19].

Here we present a new computational approach, which combines in an innovative way methods for remote homology detection techniques integrated with detailed knowledge of the original drug-target interactions to identify potential new targets within a selected genome and potential drugs to interact with those targets. These potential drugs were prioritized for phenotypic screening to verify *ex vivo* activity. We have applied this methodology to schistosomiasis to identify potential new targets and approved drugs and verify the efficacy of these inhibitors against all three human infective life stages of *Schistosoma* species. In addition, we have elucidated the *S*. *japonicum* kinome completing the kinomes of all three major infective *Schistosoma* species of humans. This, in combination with our repurposing pipeline, has allowed the identification of 22 novel schistosome potential targets, such as CHK1, CDC2, WEE, PAKA, MEK1, ABL and suggest approved drugs that could inhibit them. These include previously suggested schistosomicidal agents such as bosutinib, dasatinib, and imatinib as well as newly identified drugs such as vandetanib, saracatinib, tideglusib, alvocidib, dinaciclib. Additionally, their primary and secondary targets are also suggested and are important for maintaining the parasite’s lifecycle.

## Results

### Repurposing approved drugs as novel schistosomicidal agents

**Fig 1:**
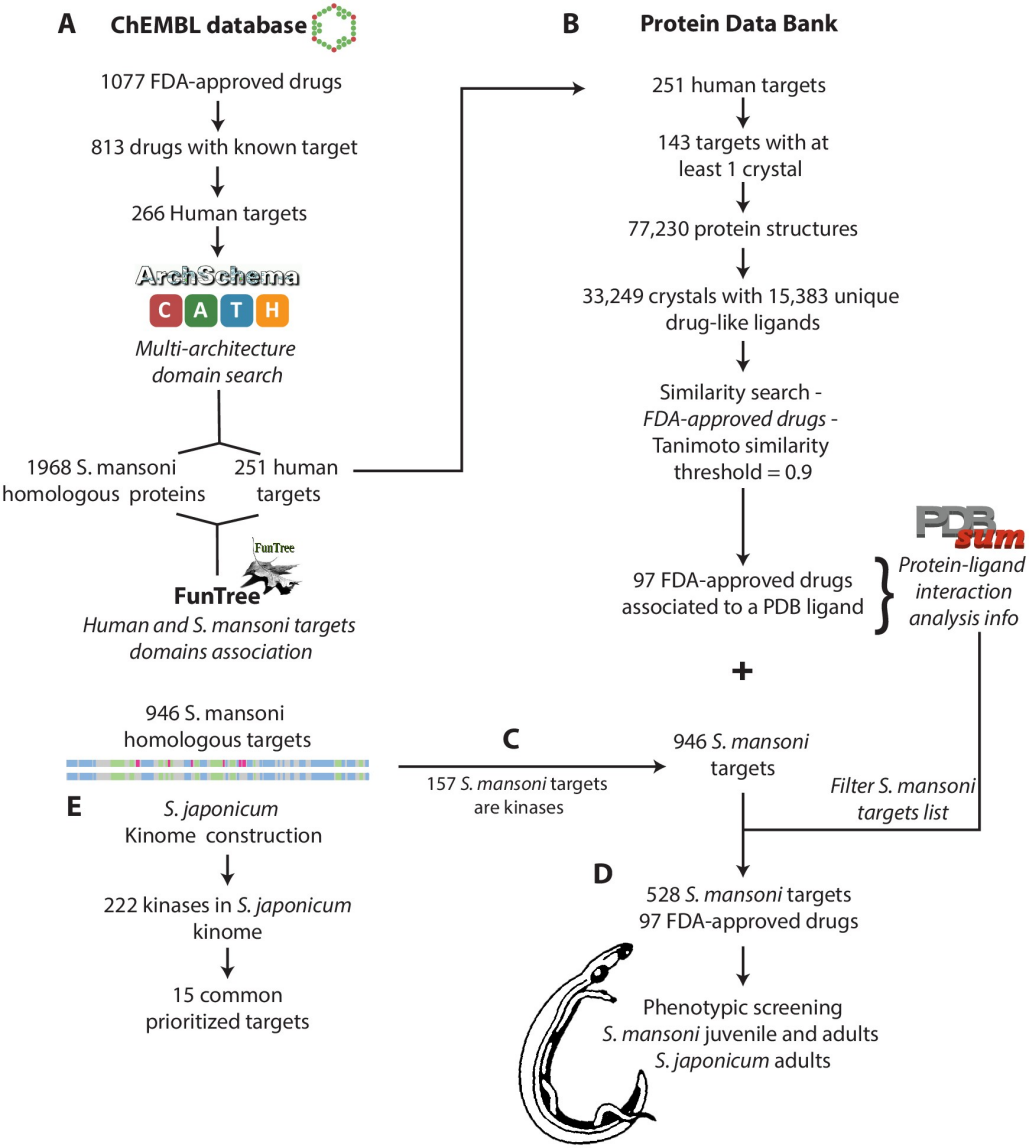
Overview of the drug repurposing pipeline. (A) New targets were identified in Schistosoma spp. based on domain homology from targets of approved drugs in humans. (B) Experimental structural information of co-crystallized drugs and drug analogues used to identify key residues involved in drug-target interactions. (C) This latter information was used to filter the results of workflow (A). (D) Drugs whose targets were selected were screened using a phenotypic screening against all three human infective life stages of Schistosoma. (E) As many of the filtered targets were protein kinases, we decided to the elucidate and classify the S. japonicum kinome.

The repurposing pipeline is summarised in Fig 1. Using the ChEMBL database, 1,077 drugs were retrieved that were identified as a ‘marketed drug’ and of the drug type ‘synthetic small molecule’. Of these, 813 drugs could be associated with a known target. The 266 unique UniprotKB identifiers of these targets were then collected. The difference between the number of associated targets and the unique identifiers is due to the sets of drugs acting on the same target. The great majority of these targets were human proteins (80%), with most of the remaining compounds targeting proteins in a variety of bacteria, viruses and other pathogens e.g. *Mycobacterium tuberculosis*, the causative agent of tuberculosis. There were two identified targets that belong to *S*. *mansoni*: a high-voltage activated calcium channel Cav B1 subunit (UniprotKB ID: Q95US7) and Cav B2 subunit (UniprotKB ID: Q962H3), both of which are listed as targets for praziquantel. No targets from *S*. *haematobium* or *S*. *japonicum* were found.

Multi-domain architectures were assigned to each of the target proteins with 194 CATH domain superfamily assigned to 251 targets and 376 Pfam domains assigned to the entire 266 targets. Focusing on *S*. *mansoni* as the major species responsible for the disease, a similar process of multi-domain architecture assignment was made, with 160 CATH domain superfamilies assigned to 1,561 proteins and 194 PFam domains assigned to 1,010 proteins. Using these domain assignments, a total of 1,968 *S*. *mansoni* proteins were identified as potential drug repurposing targets based on the occurrence of either a CATH or PFam domain found in the original drug target. As the drug targets often contain domains other than those involved in the drug interaction the number of potential repurposing targets is significantly inflated, thus further filtering is required to identify only those potential targets that contain the correct domain combinations involved in drug interactions.

The annotations and nomenclature found in the published structures in the PDB do not make it easy to distinguish drug or drug-like molecules from solvent or other crystallographic crystallization aid. We therefore manually curated a list of 326 heterogeneous atom record identifiers including all metals, ions, peptides, nucleotides and the most common crystallization agents and solvents. We used this list to filter the 77,230 protein structures retrieved from the PDB that contained a ligand to obtain 39,249 structures with 15,383 unique ligands.

Of the 252 drug-targets obtained above, 143 had at least one known crystal structure and the 39,249 structures from the PDB were filtered to find all target structures which had a drug like ligand co-crystallized. For each structure associated with a target, the SMILES notations for all ligands were obtained. They were then clustered against the known drug using Small Molecular Sub-graph Detection (SMSD) with a Tanimoto coefficient of above 0.9. This allowed for identifying structures not only that had been co-crystallized with the drug under investigation but also very similar drug analogues. Using the filtered list of structures with drug and drug-like compounds co-crystallised we could identify 97 FDA approved drugs which shared a protein domain (defined from their association with a known structure) with a domain found in a *S*. *mansoni* protein. In total 964 homologous proteins were identified in *S*. *mansoni* that could be potential targets. All these potential targets share a domain with the original drug target, however for some of them the common domain is not involved in the drug interaction. To overcome this, we used the original drug interactions obtained from PDBSum and the conservation of the interacting residues to further filter the *S*. *mansoni* potential target list to those that contained domains that could be directly involved in a drug interaction. The resulting list contained 528 *S*. *mansoni* potential targets. The ratio of approximately one drug to six targets is broadly in line with expectations based on the number of off-target interactions of compounds in humans [30]. The drug molecules identified have a broad range of indications, dominated by kinase inhibitors, but also including but not limited to antithrombotics, antihypertensives and antibacterials. A full list is provided in S1 Table. The potential targets are identified without consideration for their essentialness to the survival of the parasite to provide a list of potential targets as wide as possible. To overcome this limitation, we combined target predictions with phenotypic screening to assess the drug’s potential.

### Experimental validation

The 97 FDA approved drugs that could be considered for repurposing were used in a range of phenotypic screens against different stages of the parasites left cycle. Of the drugs that could be commercially sourced an image-based high content screen [31] was performed against the larval schistosomula using a single dose of 10μM. The drug was considered a hit if the phenotype and motility score fell below the define threshold of <-0.15 phenotype and <-0.35 motility. The compounds that produced a larval hit were subjected to a second phenotypic screen on juvenile worms, again at a single dose of 10 μM. Hits were considered as drugs that killed 70% or above of the worms by day 5. These hits were used in a final single dose screen on the adult worms, again with a hit considered as one that caused 70% mortality or above of the worms by day 5. For these hits, IC_50_ were calculated for larval, juvenile and adult worms. Larval IC_50_ were generated from average inhibition of the HCS phenotype/motility scores while the juvenile and adult IC_50_ values were replicated by visual assessment across the dose range. Adult IC_50_ values were also calculated against *S*. *japonicum*. A summary of the screen results and IC_50_ values can be found in Table 1.

**Table 1:** Summary of phenotypic screening results. A summary of the top hits from the phenotypic screening of larval, juvenile and adult *S*. *mansoni* worms as well as adult *S*. *japonicum* worms for those hits that were successful in *S*. *mansoni*. Screen hits are shown, along with motility (as a percentage) and IC_50_ values (uM). The High Content Screening (HCS) larval assays were parametrised with positive controls with Oltipraz to define phenotype and mobility values with average scores of -0.66 for phenotype and -0.92 for mobility. The juvenile and adult assays followed a previously reported method which included appropriate positive controls (see Methods). To minimise animal usage, only where a ‘hit’ was obtained further screening was performed, starting with larval progressing to juvenile and then adult for *S*. *mansoni* then following up for adult *S*. *japonicum*.

Compound name	Larva HCS Hit 10uM	Juvenile Hit Day 5 10uM	Juvenile Motility	Adult Hit Day 5 10uM	Adult Motility	Larva IC50 (HCS) (uM)	Larva IC50 (Visual) (uM)	Juvenile IC50	Juvenile IC50 (Reproduced)	Mean Juvenile IC50 (uM)	Adult IC50 (uM)	Adult *S*. *Japonicum* IC50 (uM)
Bosutinib	HIT	HIT	95.8	HIT	87.5	10.49	10.39	0.7977	0.9552	0.88	1.84	7.092
Crizotinib	HIT	HIT	78.6	HIT	70.8	6.45	6.49	3.268	1.347	2.31	3.336	N/A
Sunitinib Malate	HIT	HIT	83.3	N	62.5	4.95	6.37	4.595	2.01	3.30	3.703	N/A
Nilotinib	HIT	HIT	75	HIT	91.7	6.57	10.09	6.455*	4.648	4.65	3.924	N/A
Dasatinib	HIT	HIT	90	HIT	70.8	4.66	4.94	1.542	1.303	1.42	4.594	5.266
Imatinib mesylate	NON_HIT	HIT	92.9	HIT	75.0	15.21	15.63	5.713	4.436	5.07	8.124	19.03
Alendronate Sodium	NON_HIT	HIT	79.2	N	62.5	>25	>25	1.388*	7.201	7.20	>20	N/A
Tamoxifen Citrate	HIT	HIT	95.8	N	29.2	3.73	4.07					
Ibandronate Sodium Salt	NON_HIT	HIT	89.3	N	16.7	>25	13.01					
Triclosan	HIT	HIT	85.7	N	45.8	3.75	4.46					
Toremifene Citrate Salt	HIT	HIT	75	N	0.0	3.40	3.36					
Lapatinib	HIT	N	37.5									
Diethylstilbestrol	HIT	N	25									
Risedronate Sodium	NON_HIT	N	58.3									
Atorvastatin Calcium	NON_HIT	N	41.7									

### Extending identification of novel kinase targets

One of the main classes of compounds that emerged from our drug repurposing approach were kinase inhibitors. This is not surprising given that they represent a significant proportion of approved drugs and the importance of kinases in regulating cell processes. Currently, the kinomes have been defined for two of the three main *Schistosoma* species that infect humans. To provide a complete analysis of kinases across all three species and to use this to identify novel kinase targets we built the kinome of *S*. *japonicum* as well as updated and compared the kinomes of *S*. *mansoni* and *S*. *haematobium* with the human kinome.

The predicted proteome of *S*. *japonicum* contains 12,743 sequences, out of which we could identify and classify 165 protein kinases using Kinannote, representing 1.3% of this organism’s proteome. After thorough manual annotation and data integration using different strategies, we could increase this number to 222 identified kinases or 1.7% of the proteome. The proteomes of *S*. *mansoni* and *S*. *haematobium* contain respectively 1.9% [26] and 2% [28] kinase proteins. Further updates in *S. mansoni [27]* guided to the identification of about 2.3% kinase proteins encoded by its proteome. Our results show that the kinome of *S*. *japonicum* is composed of proteins belonging to the nine major eukaryotic protein kinase (ePK) groups. Table 2 shows an overview of the draft and final *S*. *japonicum* kinome classification, including the number of proteins added by the described pipeline.

**Table 2:** Summary of *S*. *japonicum* kinome, from its initial classification to the final classification of protein kinases.

Kinase groups	Draft	Final	Manually added
AGC	22	27	5
CAMK	26	33	7
CK1	7	8	1
CMGC	35	41	6
Other	23	35	12
RGC	1	4	3
STE	17	23	6
TK	19	31	12
TKL	8	13	5
PKL/aPKs	7	8	1
Total	165	222	58

**Fig 2:**
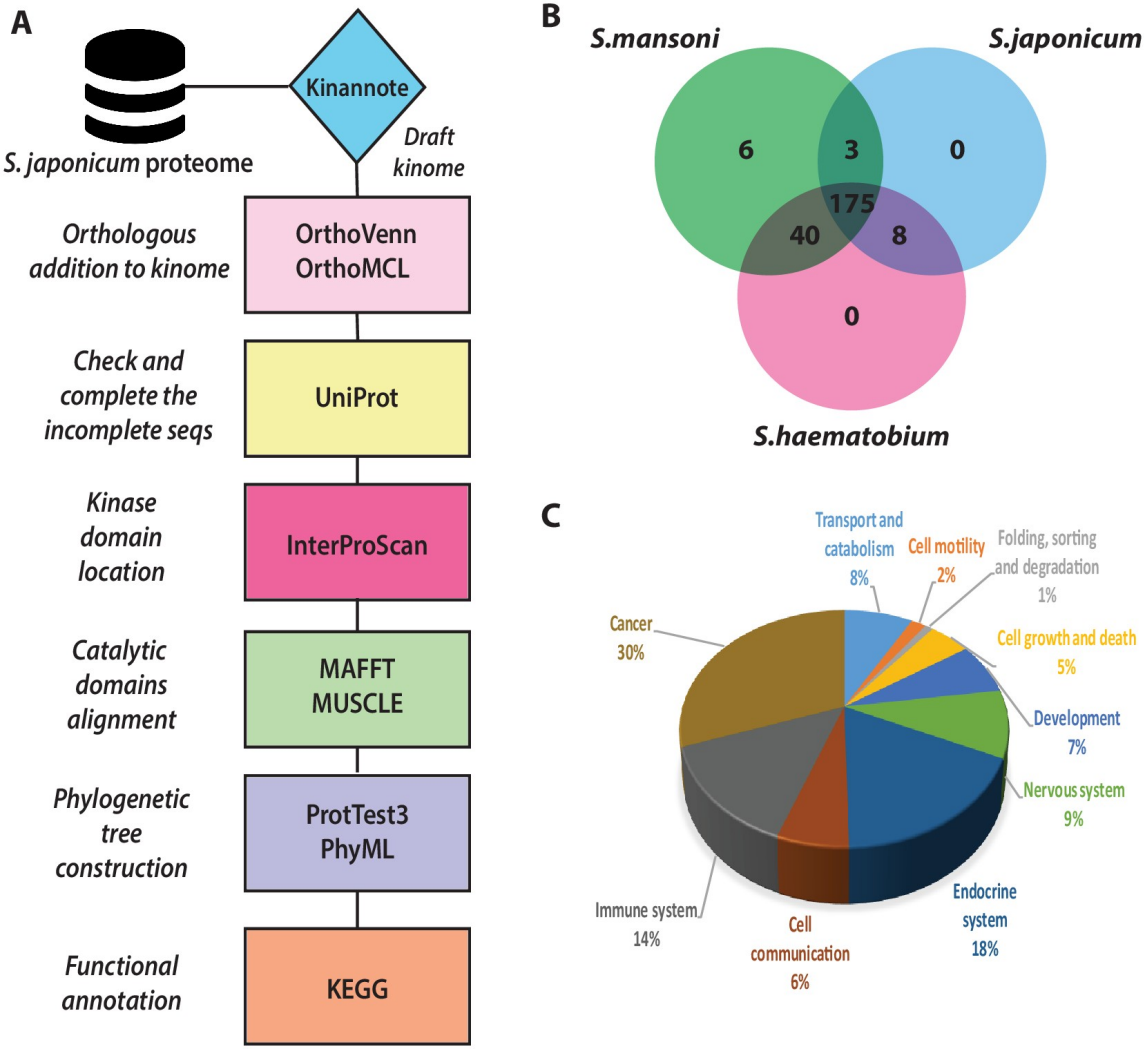
(A) General pipeline for the *S*. *japonicum* kinome construction. (B) Venn diagram displaying the overlap of proteins clusters between the three most clinically important species of *Schistosoma*. (C) Final analysis of functional annotation step: cellular processes in which *S*. *japonicum* kinases are enrolled.

Our pipeline (see Fig 2A) allowed the classification of 222 protein kinases into eight major ePKs groups and, among them, seven proteins were assigned only at “group” level, 103 at “family” level, and 111 at subfamily level (see S2 Table for the annotation details of the *S*. *japonicum* kinome). Phylogenetic trees for the major eight kinase families were constructed, and the positioning of the proteins within each group also reflects their further sub-classification into families and sub-families, and their complex interrelatedness (Fig 2B). Regarding the role of the identified kinases, functional annotation gathered from KEGG database revealed that most of them are involved in cellular processes such as cell communication, cell growth and death, and cell motility and development (Fig 2C).

The elucidated, classified and annotated *S*. *japonicum* kinome can be summarized as follows and visualised in S1 Fig. The CMGC group, which includes mitogen-activated protein kinases (MAPK), cyclin dependent kinases (CDKs) and kinases involved in splicing and metabolic control, was the largest group represented, with 34 protein kinases. From these, 13 kinases belong to the CDKs family, 10 to MAPKs, six dual-specificity tyrosine-regulated kinases (DYRKs), four CDC-like kinase (CLKs), two glycogen synthase kinases (GSKs), CDK-like (CDKLs) and SRPK, and one member belonging to CK2 family. The CAMK (Calcium and Calmodulin-regulated kinases) group was represented by CAMKs, CAMK-related kinases, MAPKAPK (MAP kinase activated protein kinase), and DAPKs (death-associated protein kinases). Among tyrosine kinase (TK) group, 15 proteins were represented by RTKs (receptor tyrosine kinases) and 13 by CTKs (cytoplasmic tyrosine kinases) and three members were not classified at family level. The AGC group comprised 27 proteins, including PKAs, PKCs, PKGs, DMPKs and RSKs. The STE group had 23 members, comprising 14 members of STE20 (MAP4Ks), 5 members of STE11 (MAP3Ks) family, and 4 members of STE7 (MAP2Ks) family. There were 13 proteins assigned to tyrosine kinase-like (TKL) group and belonging to families such as LISK (n = 2), RAF (n = 3), LRRK (n = 2), MLK (n = 2), and STKR (n = 5). Eight proteins were assigned to CK1 group, including VRK (vaccinia-related kinase) and TTBK (Tau tubulin kinase) families. Furthermore, our annotation also assigned 7 proteins to PKL group (ABC1 and RIO families). Four proteins classified into receptor guanylate cyclases (RGC) group, the smallest kinase group in *S*. *japonicum* kinome. Thirty-five proteins that did not belong to any of the major kinase groups were placed into “Other” including AUR (aurora kinase) and PLK (Polo-like kinase). A single protein was classified as an atypical kinase, assigned to PIKK family. This protein (sequence accession Sjp_0039160) has no orthologs in neither *S*. *haematobium* nor in *S*. *mansoni* [28] and in a BLAST search against WormBase (https://parasite.wormbase.org/index.html) one can find it classified as a PIKK protein in *Echinococcus multilocularis*, a cestoda worm responsible for echinococcosis.

Subsequently, we developed a target prioritization workflow using a topological network analysis. A protein-protein interaction network was constructed based on evidence gathered from *S*. *mansoni* orthologs deposited in STRING database [32]. Proteins were organized into modules according to indices of topological centrality, allowing the characterization of their biological importance.

The combination of at least two centrality measures has been proposed as a suitable strategy in defining essential genes in biological networks [33]. In biological terms, a betweenness centrality metric can indicate the relevance of a protein considering its capability of connecting different parts (processes) of the network. Considering that kinases are especially involved in signalling processes, this metric is relevant to our work. In the same way, the closeness metric, applied in a biological context, can be used as a measure of how fast the information from one node can spread to other nodes in the network, which is also of interest in the signalization context. Subgraph centrality analysed “closed walks” in the network, i.e., the importance of the protein in subnetworks, allowing better ranking in comparison to the whole network and better discrimination among the proteins [34] S2 Fig. Using this approach, we identified a list of 26 protein kinases highly relevant for the interaction network generated from STRING database [32], that could be prioritized as new potential drug targets.

## Discussion

The identification of novel indications for existing licenced therapeutics offers an attractive route for neglected topical diseases drug discovery, with the potential to reduce development time and costs and decrease the likelihood of failure in clinical testing. Thus, we developed a novel computational repurposing pipeline (summarised in Fig 1) exploiting conserved drug-target interaction properties in distantly evolutionary related potential targets. Predictions were supported by phenotypic screening on different infective life stages and species of *Schistosoma*.

The top hits from the phenotypic screening are from a series of tyrosine kinase inhibitors developed as first line (imatinib) and second line (bosutinib, crizotinib, nilotinib, dasatinib) anti-cancer treatments for chronic myelogenous leukemia (CML). Imatinib has already been identified as a possible drug-repurposing candidate based on the homology between the human gene target and a gene in the *Schistosoma* genome during its annotation [35–37]. The second line treatments have recently been identified from larval and follow-up adult phenotypic screening [38]. These independent observations validate the approach used here. Though many of our hits agree with previous studies some, such as nilotinib, show different *ex vivo* activity in contact to previous findings [39]. Differences between screens have been observed before [17] and does not mean that either are incorrect: differences in strains used, concentrations of compounds, duration of assessment, DMSO concentrations, if the adults were paired or un-paired, and if un-paired if they were all male, all female or mixed can all have an effect.

**Fig 3:**
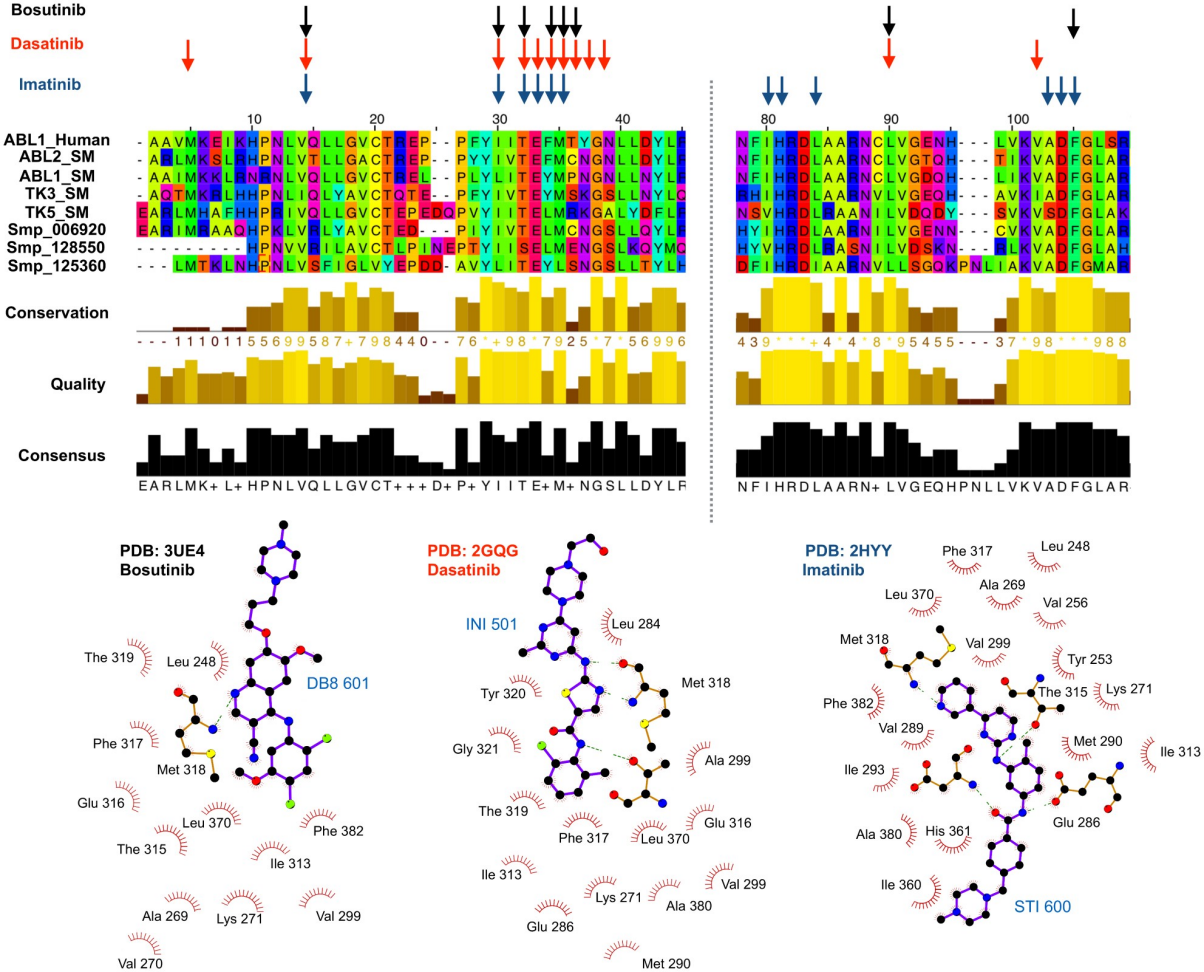
Alignment and conservation of key residues involved in new targets to tyrosine kinase inhibitors. The key residues involved in drug/target interactions (shown bottom) are highlighted by arrows in structurally informed multiple sequence alignment (using Taylor conservation colouring) for the potential repurposing targets identified in *Schistosoma mansoni*. Three tyrosine kinase inhibitors are shown: Imatinib (blue), Dastinib (red) and Bosutnib (black).

**Fig 4:**
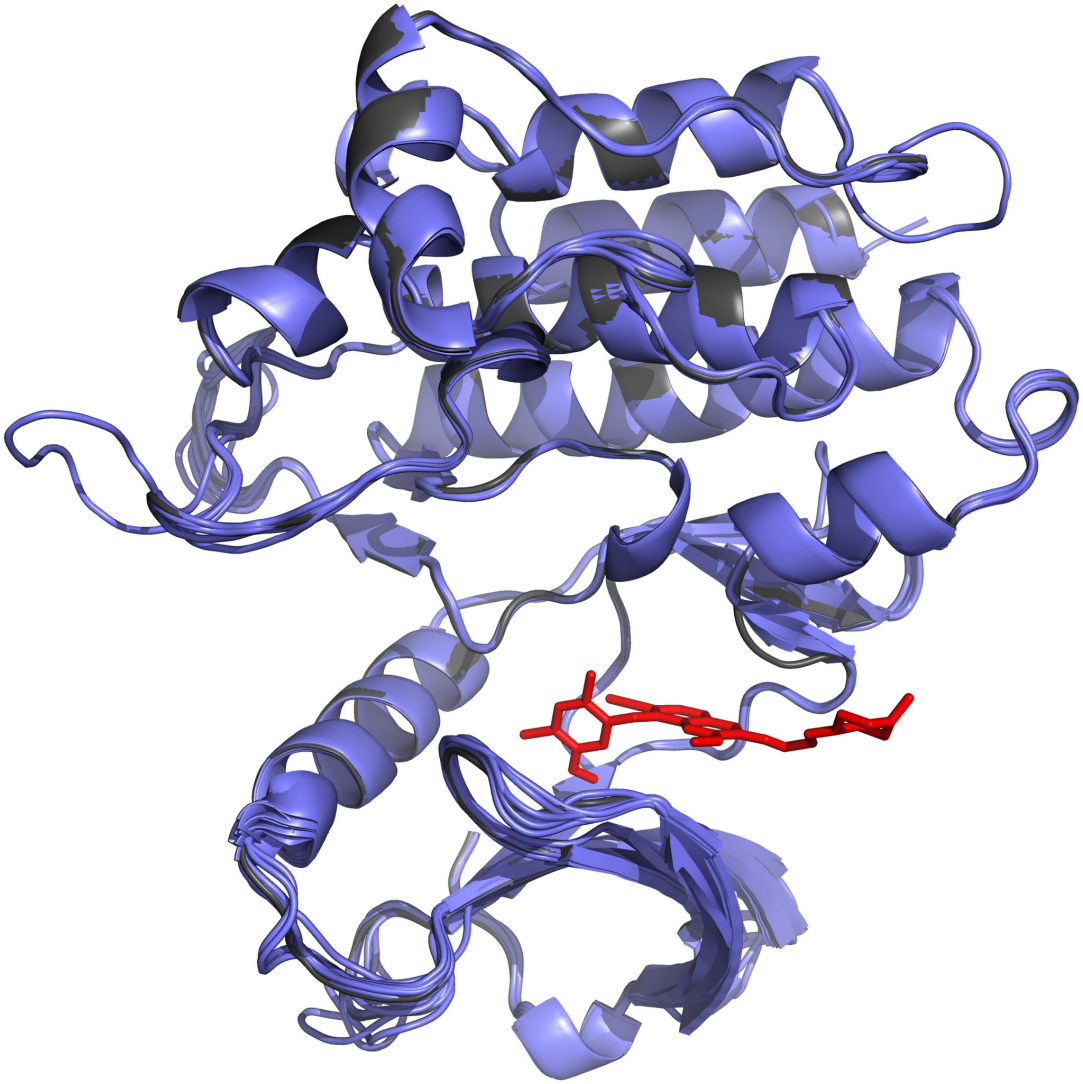
Modelling targets of imatinib. Structural models of the targets (ABL1, ABL2, TK3_SM, TK5_SM, Smp_006920, Smp_128550, Smp_125360) identified in *Schistosoma mansoni* (shown as cartoon in blue) of imatinib, which is docked to the targets (shown as sticks in red).

Though the potency of these compounds is promising, they are not immediately of the quality required of a lead compound or directly transferable to the clinical setting. However, they provide excellent starting point to begin to explore variations of the compound within the same chemical series. As we have knowledge of the potential targets of these compounds, including the ability to generate molecular models, it will be possible to explore *Schistosoma* specific features of the proposed targets to design compounds with higher potency using ligand-based and structure-based drug design. It is important to note that the top hits showed potency against all three life-stages, including juvenile worms that is a known short coming of PZQ activity [40, 41]. By using a combination of target-based remote homology detection and phenotypic screening, this dual method can provide more information than either of the two approaches separately. This includes the detection of several potential targets for these compounds beyond the two genes annotated as homologs of the human target gene (see Fig 3). This comprises several proteins that are, as yet, not annotated with any function. In addition, by using a structure-based approach, structural models of the new potential targets can be predicted and docking can be performed with the repurposed compound (see Fig 4). This allows for exploration of novel binding modes and elaboration or virtual screening of analogue compounds to exploit features that are pathogen-specific. Though phenotypic screening remains a gold standard for assessing if the potential re-purposed drugs are effective against the worm, it would be possible to integrate other sources of data that hold information relating to how essential the potential target might be to the parasite. This could be introduced as a further triage step in the computational pipeline.

**Fig 5:**
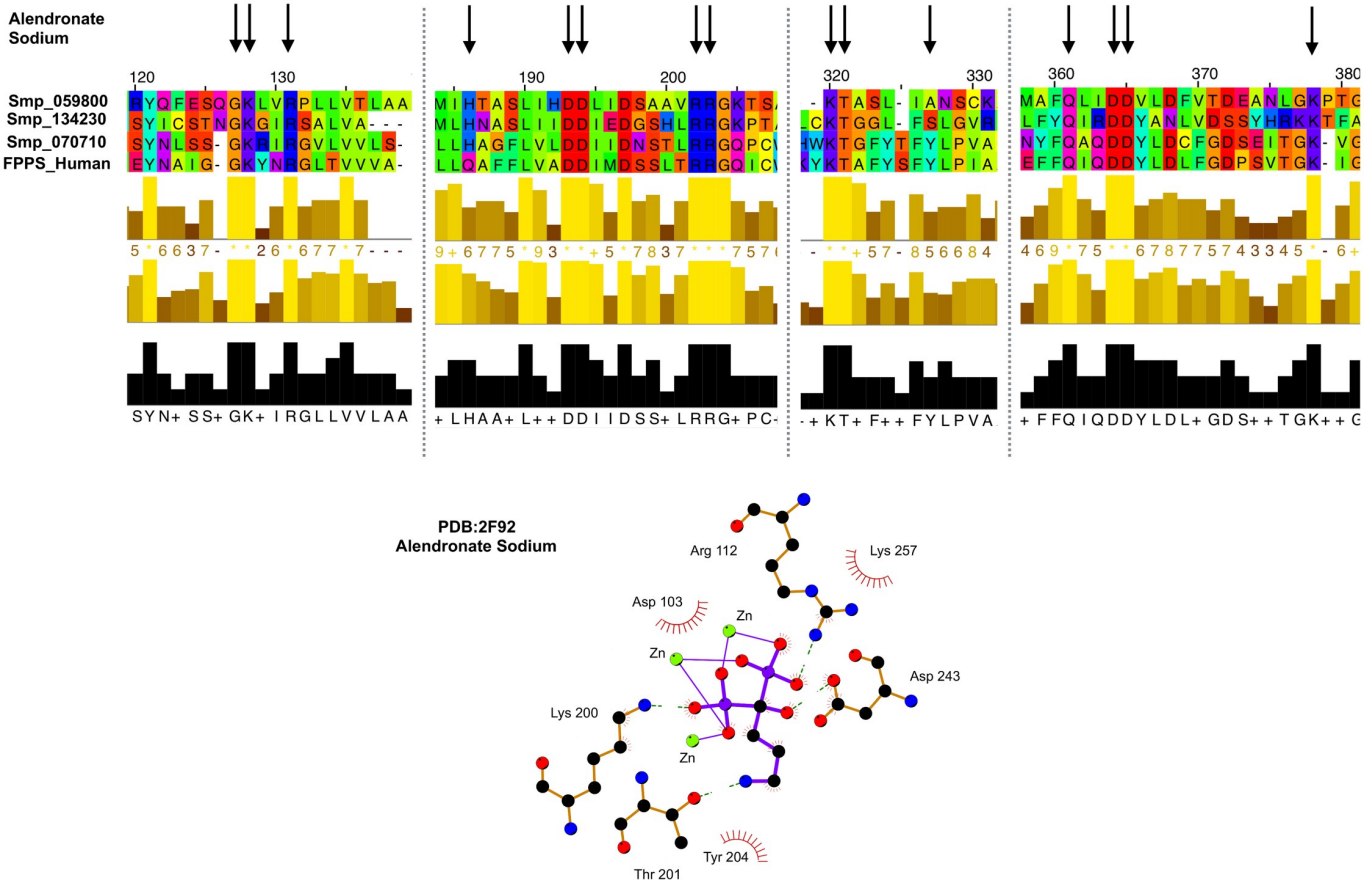
Alignment and conservation of key residues involved in new targets to alendronate sodium. The key residues involved in drug/target interactions (shown bottom) are highlighted by arrows in structurally informed multiple sequence alignment (using Taylor conservation colouring) for the potential repurposing targets identified in *Schistosoma mansoni*. The residues involved in drug interactions are spaced across the alignment, therefore only the relevant sections of the alignment are shown for clarity.

Only a relatively small fraction of the suggested repurposed compounds lead to success with all three infective life stages in phenotypic screening. For example, sodium alendronate, an osteoporosis treatment that targets farnesyl diphosphate synthase, has many of the residues involved in drug interaction conserved in three homologues identified by the pipeline (see Fig 5). Also, the drug has proved success in assays against farnesyl diphosphate synthase in *Leishmania donovani*. However, in phenotypic screening it did not produce a hit. This could be due to low bioavailability of the drug in the worm or the lack of expression of the target. This demonstrates the necessity of using a combination of target-based approaches with phenotypic screening.

As many of the predictions were kinase inhibitors, we also undertook an extensive and though investigation of the kinomes of all three infective *Schistosoma* species, including the yet unexplored kinome of *S*. *japonicum*, to suggest new targets and novel schistosomicidal agents. Among the obtained list of prioritized 26 protein kinases, the CAMK group was the most represented, with four CAMKL family members and one PIM family member, followed by the AGC group, with one member in each family RSK, PDK1, SGK, PKA, and PKG. The group “Other” was also represented by five members of families AUR, PLK, TTK, WEE, and NAK, while CMGC and STE groups were represented by four proteins, belonging respectively to families CDK, MAPK, and GSK; and STE7, STE11, and STE20, respectively. The smallest group was TKL with two members of families LRRK and RAF.

After predicting these kinases as potential drug targets, we interrogated drug databases to search for drugs that could interact with one or more of these potential targets. Table 3 shows the sample information about five of these kinases; additional information, including approved drugs for identified essential kinases and prioritized potential targets list is available in S3 Table.

**Table 3:** Top 5 prioritized kinases as potential schistosomicidal targets and its human identity (%).

ID	Classification	Functional annotation	Human identity (%)	Kinase SARfari	
				Bioactivites^a^	Number of compounds
**Sjp_0031130**	CMGC/CDK/CDC2	Cell growth and death	66	2971	2531
**Sjp_0035520**	CMGC/MAPK/ERK1	Cell growth and death	57	1068	1417
**Sjp_0046880**	Other/AUR	Cell growth and death, oocyte meiosis	45	1913	1613
**Sjp_0054140**	Other/PLK/SAK	Signal transduction	36	196	167
**Sjp_0026020**	CMGC/GSK	Signal transduction	73	3449	2744

^**a**:^ available screening data related to each protein kinase that was found at Kinase SARfari online platform (https://www.ebi.ac.uk/chembl/sarfari/kinasesarfari)

Of those 26 kinases prioritized after the centrality analysis step, 15 proteins had orthologs in the list of 528 potential targets gathered from our protein domain based approach for drug repurposing. Of those 15 *S*. *japonicum* kinases, 5 kinases were assigned only to lithium citrate, an approved drug for treating psychotic disorders; 10 kinases were assigned to 16 approved drugs, including bosutinib, dasatinib, and imatinib, which were the best experimentally validated hits in our phenotypic screening assays. The protein kinases involved in interactions with those drugs are: CMGC/CDK/CDC2; STE/STE20/PAKA; CAMK/PIM; CMGC/MAPK/ERK; CMGC/MAPK/p38; CMGC/GSK; AGC/PKA; CAMK/CAMKL/NIM1; Other/AUR; Other/TTK. Some representing kinases of those prioritized groups, were identified as possible targets of bosutinib, dasatinib, and imatinib, are well as some kinases well characterized in the literature, such as PKA, PLK, p38, and ERK. The Other/AUR kinase is more related to trypanosomatid protozoan parasites [42, 43]. Therefore, this kinase is suggested as one of the novel biological targets for killing *Schistosoma* genus parasites.

Cyclic AMP (cAMP)-dependent protein kinase/protein kinase A (PKA) have been mapped in both male and female worms and localized in its activated state. A consistent correlation has been established between PKA and tegument, somatic musculature, seminal vesicle and gynaecophoric muscles [44]. Polo-like kinases (PLK) represent a conserved group of cell-cycle regulators and, in *S*. *mansoni*, PLK1 and Sak kinases have been described as a confirmed gametogenesis regulator [45–47] and an important protein in gonad maturation [47] respectively.

ERK kinase is a validated target in *S*. *mansoni* [48]. The effects on worm coupling, reduction of egg release, and worm homeostasis were pronounced [45, 46, 49]. A connection between PKC and ERK signalling has been observed [49], turning these proteins into interesting points of intervention in the context of schistosomiasis treatment. Mitogen-activated p38 kinase is a highly conserved protein kinase in different species, described in *S*. *mansoni* with a key role in transition between two life stages of this trematode [50]. This highlights the enormous importance of kinase proteins as biological targets in the search of new treatments for schistosomiasis.

A limitation of our *S*. *japonicum* kinome elucidation approach include the use of a reference *S*. *japonicum* genome (and, consequently, proteome) that cannot be considered a definitive picture of all genes and proteins coded by *S*. *japonicum*. The *S*. *japonicum* genome described by Zhou et al. [51] that we used during the determination of the kinome repertoire was sequenced using a traditional Sanger instrument in 2009 and, while that usually associates to good base qualities, the throughput obtained is much lower than current NGS instruments. Using evidence from full length cDNAs (flcDNAs) and expressed sequence tags (ESTs) available then, the authors reported a coverage >90% of their published *S*. *japonicum* genome. As such, it is possible that we may have missed some kinases that could not be predicted using our approach because the underlying sequences were not present in the proteome reference used.

Through the development of a new computational pipeline to suggest novel indications for currently approved drugs and supported by phenotypic screening, several new targets and drugs have been suggested as starting points in the development of new schistosomicidal agents. As many of these new targets and drugs are kinases and kinase inhibitors, we undertook a detailed and extensive comparative analysis of the kinomes of all three human infective Schistosoma species and the human host. This allowed the identification of kinase targets and existing and novel inhibitors that could be effective across all three Schistosoma species. The computational drug repurposing pipeline is generalizable, as it only requires a change in the target genome identifier for it to be applied to other infectious disease agents.

## Materials and methods

### Ethics statement

Experimentation was carried out using the NC3Rs and ARRIVE guidelines under the United Kingdom Animal’s Scientific Procedures Act 1986 with approval from the London School of Hygiene and Tropical Medicine Ethics committee. Male CD1 mice (aged 5–6 weeks) were bred on site using SPF conditions with access of food and water *ad libitum*.

### Drug repurposing pipeline

A computational drug repurposing pipeline was developed to predict potential drugs that could be repurposed as antischistosomal compounds. An overview of the pipeline is shown in Fig 1. Data for all currently FDA approved drugs and their known molecular targets was collected from ChEMBL database v. 18 [52]. The multi-domain architecture for these drug-targets was defined using a combination of CATH-Gene3D [53] and Pfam [54], via ArchSchema [55]. The same process of multi-domain architecture definition was conducted on the non-redundant set of *Schistosoma mansoni* proteins in UniprotKB [56]. CATH structure based domains were clustered using the agglomerative clustering tools associated in the alignment generation steps used in FunTree [57] (Fig 1A). This generates an association by individual protein domain between current drug targets and equivalent domains in protein from *S*. *mansoni*.

Concurrently, using the target associations obtained from ChEMBL, any known crystal structures were collected from the Protein Data Bank (PDB) [58], removing structures that only contain ions, metals, crystallographic solvents or aids as ligands (Fig 1B). A list of the heterogeneous atom records used in this exclusion can be found in S4 Table. For each target, the remaining structures were clustered with the FDA-approved drugs based on the similarity to the co-crystalized ligands using Small Molecular Sub-graph Detection (SMSD) [59] with a similarity threshold of 0.9 computed using the Tanimoto coefficient (Fig 1B). This allowed for similar drug analogues to be picked up where the actual drug had not been co-crystallized. Automatic structural analysis to identify the residues within the structure responsible for the interaction between the ligand and its target was identified using PDBSum [60].

The ligand-target interaction information was combined with the domain similarity clustering (Fig 1A and 1B) to remove domains that were not involved with the interaction and to filter for those domains where these specific residues were conserved. For each target, a list of proteins in *S*. *mansoni* that contained a homologous domain where there is conservation of ligand binding residues could be retrieved. These represent the potential repurposed drug targets (Fig 1C). Based on this association, molecular structural models of the domains could be automatically generated using MODELLER [61]. The drugs whose targets were selected by this pipeline were screened using a phenotypic screening strategy against all three human infective life stages (Fig 1D).

### Construction of the *S*. *japonicum* kinome

As a first step towards defining the *S*. *japonicum* kinome, we downloaded its proteome from the GeneDB database (http://www.genedb.org/Homepage/Sjaponicum). The kinomes of *S*. *mansoni* [26] and *S*. *haematobium* [28] were used as sources of orthologs to compare the protein kinases of *S*. *japonicum*. Weight was placed on orthologs from *S*. *mansoni* that is phylogenetically closer to *S*. *japonicum* than *S*. *haematobium*. This approach was complemented by using the Kinannote software [62] using the -*m* option for metazoan organisms, allowing the generation of a draft *S*. *japonicum* kinome. After this initial prediction, sequences that were not classified were manually curated and re-classified as far as possible. The work flow is summarised in Fig 2A.

Orthologous sequences were predicted through OrthoMCL [63] by pairwise sequence comparison between kinases of *S*. *mansoni* and *S*. *haematobium*, grouping *S*. *japonicum* proteins to kinases and were not classified previously by Kinannote. Using UniProt database [56] and the InterProScan webserver [64] to identify the kinase domains of each protein previously predicted and classified, and to determine a consensus localization of the catalytic domain.

### Kinome tree generation

For construction of phylogenetic trees per individual kinase family, multiple sequence alignments of the amino acid sequence spanning the consensus catalytic domains of each protein were made using MAFFT v. 7.215 in most accurate mode (L-INS-i) [65], followed by refinement using MUSCLE v. 3.8.31 (*—refine* parameter) [66]. The selection of an evolutionary model for maximum likelihood (ML) tree reconstruction was performed using ProtTest3 [67]. ML trees were generated using PhyML 3.0 [68] with 1,000 bootstrap replicates. FigTree (available at http://tree.bio.ed.ac.uk/software/figtree/) was used to edit and export the trees.

### Prioritising kinase targets

For the kinase prioritisation as biological targets, we performed an integrative analysis, using, the Kyoto Encyclopaedia of Genes and Genomes (KEGG) database [69] as a resource for functional annotation and pathway contextualization of the classified kinases with the BlastKOALA analysis tool (http://www.kegg.jp/blastkoala/). In parallel, the kinomes of *S*. *mansoni*, *S*. *haematobium*, and *S*. *japonicum* were compared using the OrthoVenn webserver [70] to group kinases into clusters and to identify the number of common clusters among these three species (see Fig 2B). Data from the STRING database [71] was used to construct a protein-protein interaction network. For this purpose, the predicted, curated, and annotated kinome of *S*. *japonicum* was used as input and the webserver automatically recovered the best hits from *S*. *mansoni* proteins deposited in the database. Then, a network of 214 orthologous kinase proteins was generated and allowed the identification of relationships among those proteins. Other relevant information about these proteins, e.g., co-expression profiles, existence of three-dimensional crystal structure, etc. were gathered to enrich the annotation. The interaction network was exported from STRING and analysed within the Cytoscape platform (v. 3.4.0, http://www.cytoscape.org/), which allows for data integration, topological analysis, and visualization [72]. Local and global centrality metrics were calculated using *cytoNCA* plugin [73] for each protein in the network and, after manual curation, only a subnetwork containing the most important proteins for the entire network was kept. In addition, we conducted searches in the DrugEBIlity portal (https://www.ebi.ac.uk/chembl/drugebility/) to gather further information about the prioritized targets, including their identity with human orthologous proteins, related bioactivity data, and drugs potentially targeting those kinases (see S2 Fig).

### Parasite maintenance and preparation

All experiments performed using *S*. *mansoni* utilized the Puerto Rican strain and was maintained through *Biomphalaria glabrata* and CD1 mice. *Schistosoma japonicum*, Philippine Strain, was maintained through *Oncomelania hupensis* subsp. *quadrasi* and CD1 mice (BEI resources, MD, USA). Schistosomula were prepared and isolated using a percoll gradient as previously described [24].

*S*. *mansoni* juvenile or adult worms were recovered by perfusion 3 or 6 weeks later respectively from mice infected subcutaneously as previously described [24]. For *S*. *japonicum* worms were generated using the same technique but with a 6 or 12-week incubation period for juvenile or adults respectively. Recovered *S*. *mansoni* and *japonicum* worms were then washed free of blood and placed into culture.

The schistosomula generated were incubated over- night in M169 supplemented with 100U/ml Penicillin, 300μg/ml Streptomycin, 0.25μg/ml Fungizone (Amphotericin B) (Gibco, UK) and 5% Foetal Calf serum. For production of S. mansoni juvenile or adult worms, mice were infected subcutaneously under mild isoflurane (Merial Animal Health Ltd (UK)) anaesthesia with, respectively 1400 (for juveniles) or 450 cercariae (for adults). Worms were recovered from infected mice using sterile techniques by portal perfusion 3 weeks (for juveniles) or 6 weeks (for adults) post-infection using warm perfusion medium (Dulbecco’s Modified Eagle’s Medium [DMEM], 2mM L- glutamine, 100 Units/ml penicillin, 100μg/ml streptomycin, 20mM Hepes, 10 Units/ml heparin [Sigma, UK]. Adult worms were washed free of red blood cells using the perfusion medium, and finally placed in culture in complete medium (cDMEM: DMEM, 2mM L-glutamine, 100 Units/ml penicillin, 100μg/ml streptomycin, 10% foetal calf serum (FCS) at 37°C, in an atmosphere of 5% CO2. Juvenile worms were sedimented successively following washing with cold perfusion medium and then suspended in cold cDMEM until dispensed to avoid attachment to the plastic tubes.

### High-content schistosomula screening assays

The larval High-Content Screening (HCS) was run as described previously [31]. Briefly cultured schistosomula were cultured for 72 hours in absence or presence of compound. After a gentle re-suspension using a Biomek FxP to generated an even distribution of parasites across the well, images were collected at 10x for phenotype analysis and at 4x over a time-series for motility analysis using an Image Xpress micro (Molecular devices, USA). Image analysis was then conducted using Pipeline Pilot 9.0 (Biovia, USA). Phenotype analysis was conducted using a previously validated two class Laplacian-modified Bayesian categorization model built using standard schistosomacides and motility analysis by individual parasite area displacement over the time series [31]. To generate larval IC50s the percent inhibition at specific compound concentrations were calculated by comparison to the mean DMSO scores (32 wells) on each plate. These were then imported into Prism 6 (Graphpad) to generate IC50 values.

### Juvenile and adult worm screening assays

For the juvenile assay 5–8 individual worms and the 3 adult worm pairs were cultured in the absence or presence of compound for 5 days [24]. Drug effects were then determined by assessing the viability of individual worms as described by Ramirez *et al* [74] and calculating the mean percentage inhibition, a hit being defined as ≥70% reduction in viability. To calculate IC_50_ against juvenile or adult worms the percent inhibition were plotted against drug concentration in Prism 6 (Graphpad). To minimise the number of animals used, only those compounds that were considered a hit were tested further to obtain IC50 values.

## Supporting information

S1 Table Summary of potential schistosomicidal repurposed drugs. (XLS)

S2 Table Draft kinome of *S*. *japonicum* and some relevant information, including ortholog proteins in *S*. *mansoni*. (XLSX)

S3 Table Prioritized kinases as potential schistosomicidal targets. (XLSX)

S4 Table A curated list of heterogeneous atom records used as part of the filter of non-drug like co-crystallised compounds from the PDB. (CSV)

S1 Fig Kinome tree of *S*. *japonicum*. All major kinase groups are represented above, except the atypical kinase group. Individual trees were constructed for each group and a human representative kinase in each group was used for rooting the tree. Black circles indicate bootstraps higher than 60%.
(PNG)

S2 Fig Potential biological targets for new schistosomicidal agents identified through the described bioinformatic pipeline. Size of each node in the left network is displayed accordingly to its Degree metric: the bigger the size, more connected the node. Blue protein-protein interaction network was gathered from STRING webserver and analyzed in Cytoscape v.3.4.0, where centrality metrics was calculated through cytoNCA plugin and a subnetwork was generated from highlighted nodes. In concordance to calculated metrics, highlighted nodes are essential for the protein-protein interaction network and, in a biological context, they were prioritized as potential biological targets for new schistosomicidal agents.
(PNG)
