# Supplementary figures and images for: Computationally-guided drug repurposing enables the discovery of kinase targets and inhibitors as new schistosomicidal agents
Source: PLoS Comput Biol. 2018 Oct 22;14(10):e1006515. doi: 10.1371/journal.pcbi.1006515 (PMC6211772; doi:10.1371/journal.pcbi.1006515)

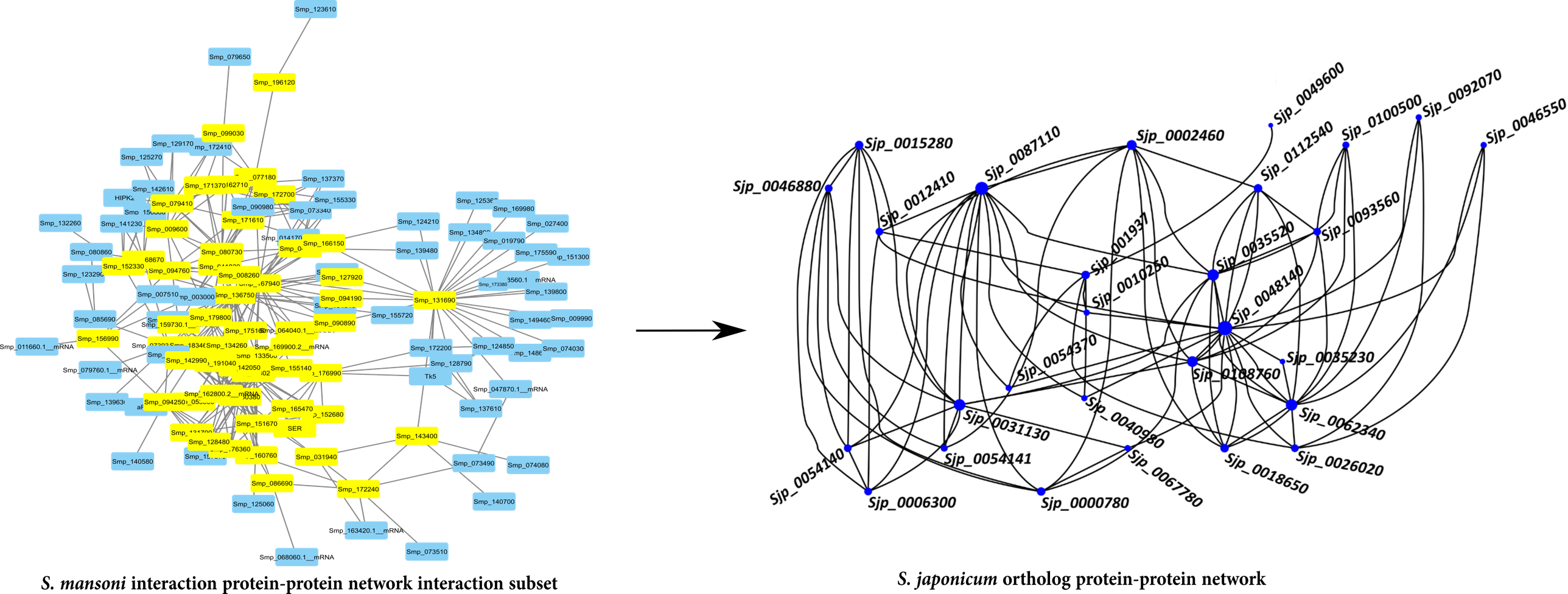

Supplement: S2 Fig — Size of each node in the left network is displayed accordingly to its Degree metric: the bigger the size, more connected the node. Blue protein-protein interaction network was gathered from STRING webserver and analyzed in Cytoscape v.3.4.0, where centrality metrics was calculated through cytoNCA plugin and a subnetwork was generated from highlighted nodes. In concordance to calculated metrics, highlighted nodes are essential for the protein-protein interaction network and, in a biological context, they were prioritized as potential biological targets for new schistosomicidal agents. (PNG) [file pcbi.1006515.s006.png]
